# Human Vection Perception Using Inertial Nulling and Certainty Estimation: The Effect of Migraine History

**DOI:** 10.1371/journal.pone.0135335

**Published:** 2015-08-17

**Authors:** Mark A. Miller, Catherine J. O’Leary, Paul D. Allen, Benjamin T. Crane

**Affiliations:** 1 School of Medicine and Dentistry, Rochester, NY, 14642, United States of America; 2 Department of Otolaryngology, University of Rochester, 601 Elmwood Ave, Box 629, Rochester, NY, 14642, United States of America; 3 Department of Bioengineering, University of Rochester, Rochester, NY, 14642, United States of America; 4 Department of Neurobiology and Anatomy, University of Rochester, 601 Elmwood Avenue, Box 629, Rochester, NY, 14642, United States of America; University of Melbourne, AUSTRALIA

## Abstract

Vection is an illusory perception of self-motion that can occur when visual motion fills the majority of the visual field. This study examines the effect of the duration of visual field movement (VFM) on the perceived strength of self-motion using an inertial nulling (IN) and a magnitude estimation technique based on the certainty that motion occurred (certainty estimation, CE). These techniques were then used to investigate the association between migraine diagnosis and the strength of perceived vection. Visual star-field stimuli consistent with either looming or receding motion were presented for 1, 4, 8 or 16s. Subjects reported the perceived direction of self-motion during the final 1s of the stimulus. For the IN method, an inertial nulling motion was delivered during this final 1s of the visual stimulus, and subjects reported the direction of perceived self-motion during this final second. The magnitude of inertial motion was varied adaptively to determine the point of subjective equality (PSE) at which forward or backward responses were equally likely. For the CE trials the same range of VFM was used but without inertial motion and subjects rated their certainty of motion on a scale of 0–100. PSE determined with the IN technique depended on direction and duration of visual motion and the CE technique showed greater certainty of perceived vection with longer VFM duration. A strong correlation between CE and IN techniques was present for the 8s stimulus. There was appreciable between-subject variation in both CE and IN techniques and migraine was associated with significantly increased perception of self-motion by CE and IN at 8 and 16s. Together, these results suggest that vection may be measured by both CE and IN techniques with good correlation. The results also suggest that susceptibility to vection may be higher in subjects with a history of migraine.

## Introduction

Vection is an illusory perception of motion that may occur when a moving visual stimulus fills the majority of the visual field [1]. The ambiguity of self- vs external-motion arises because the labyrinth cannot always provide reliable self-motion information, such as during long, constant velocity motion [2–5], making the correct interpretation of visual motion as external or as self-motion important.

Several studies have attempted to quantify vection, primarily with magnitude estimation, where the subject assigns a subjective numeric value to their perception [6–11]. This technique is simple to implement, however due to the subjective nature of the reporting it is difficult to determine if differences in subject responses are due to differences in underlying perception or to differences in the interpretation of the stimulus in relation to the reporting scale [12–14]. Furthermore, vection magnitude estimate techniques can vary between studies, making them difficult to compare [6,11,15]. These problems have been addressed in the past by normalizing responses across subjects [16], but this solution assumes all subjects had the same underlying perception, which may not be the case. Although attempts have been made to calibrate magnitude estimates based on inertial motion [17,18], this becomes problematic as sequentially presented stimuli may be difficult to match due to adaptation and working memory constraints.

Some of these problems with magnitude estimation may be overcome by using direct inertial nulling (IN), which is a technique wherein visual and inertial motions are presented simultaneously, with the modalities presented in opposition in order to determine the visual stimulus that produces perceived vection that is nulled by the inertial movement. Vection has been successfully measured in this fashion in fore-aft, lateral, and rotational studies [12,19,20], however past studies have not attempted to compare magnitude estimates with IN measurements using the same visual stimulus, which makes it difficult to compare results across studies in which only magnitude estimates were used.

The goal of the current study is to determine the effect of visual stimulus duration on the perception of vection and the origin of the substantial between-subject variation in vection perception. The first experiment uses an adaptive inertial nulling technique to determine the point of subjective equality (PSE), where the probability of a subject reporting motion in one direction or the other is equal [12]. The same range of visual motion stimuli were also investigated using a certainty estimate technique.

Serendipitously, Experiment 1 suggested that subjects endorsing migraine symptoms had stronger perception of vection. Migraine patients often suffer from vestibular symptoms, including vertigo and the feeling of tilting during migraine episodes [21–25]. Additionally, it has frequently been observed that easy motion sickness is a common feature in migraine and specifically visually-induced motion sickness (VIMS) [22,23,26–30]. Consequently Experiments 2 and 3 prospectively enrolled subjects with a history of migraine and control subjects screened for no migraine history to explore the hypothesis that a diagnosis of migraine may be an important confounding variable in vection studies and explain some of the variability in vection susceptibility.

## Methods

### Ethics Statement

Written informed consent was obtained from all participants. The study protocol and written consent document was approved by the University of Rochester Research Subjects Review Board and conducted according to the principles expressed in the Declaration of Helsinki.

### Equipment

Study measurements were performed with a six-degrees of freedom Hexapod Motion Platform (HMP) (Moog, East Aurora, NY, USA, model 6DOF2000E) connected to a visual display. This provided platform motion coupled with a visual stimulus and has been previously used in this laboratory [30,31].

**Fig 1:**
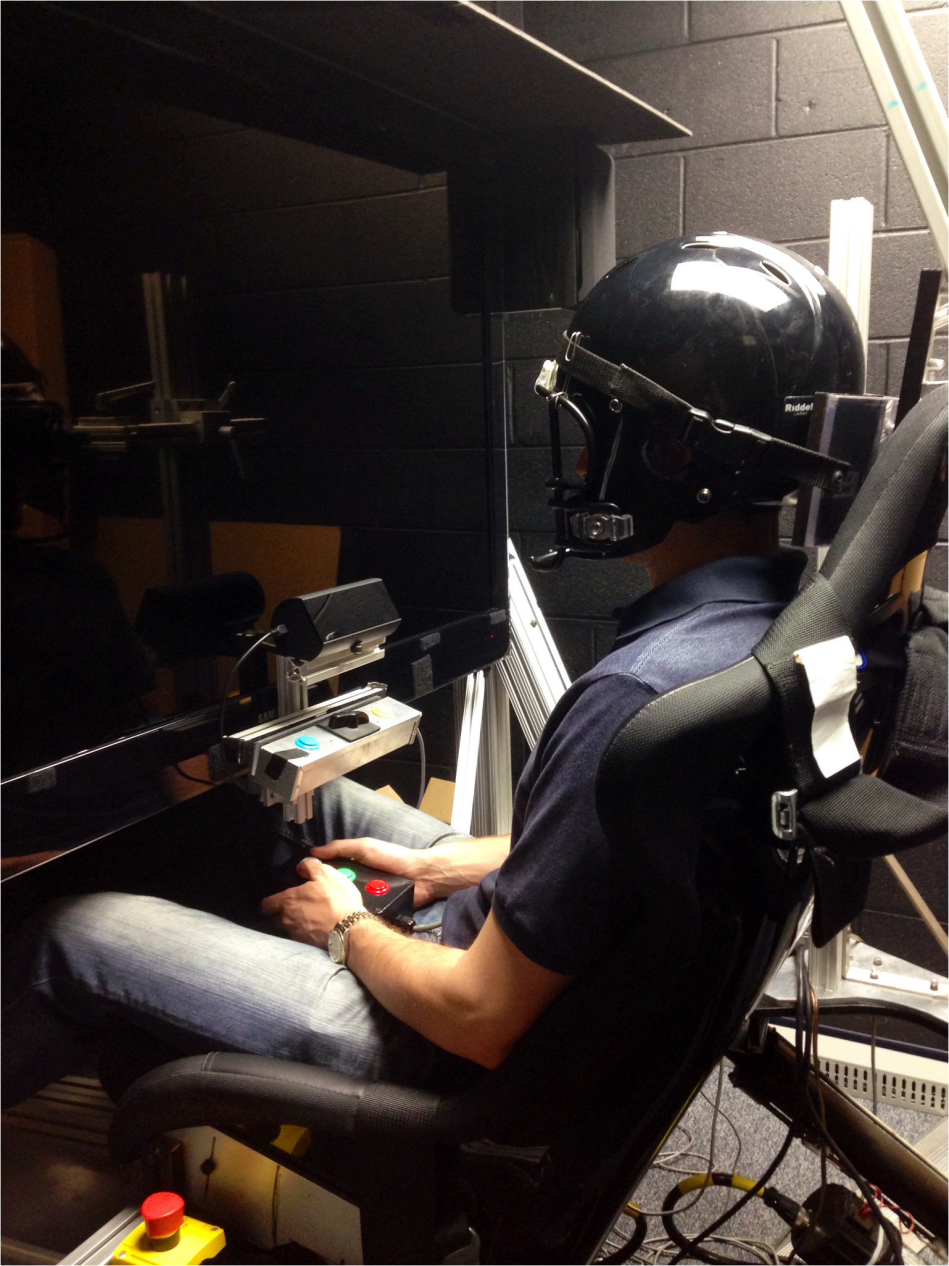
Experiment Setup.

Subjects sat upright in a padded racing seat with lumbar and seat bolsters mounted to the platform (Corbeau, Sandy UT, model FX-1). A four-point racing style harness held the body in place, and subjects wore an appropriately sized American style football helmet, with the facemask removed so as not to obscure the visual field. The helmet was fixed securely to the HMP using a custom-built structure, ensuring that head motion was closely coupled to the platform. The setup for all three experiments is shown (Fig 1).

Platform noise was masked with white noise produced from two platform-mounted speakers. The intensity of the white noise was varied during platform motion so that peak masking occurred at the time of peak motor noise. The intensity of the noise was independent of the direction of platform motion and was delivered for both PSE and CE trials.

The visual stimulus was a computer generated, three-dimensional stereoscopic image consisting of red and green triangles that simulated movement through a star-field [16]. Red-green anaglyph glasses provided enhanced visual disparity as previously described [32]. The stimulus was presented on a horizontal color LCD screen which filled 98° of the azimuth. Each star consisted of a triangle 0.5 cm in height and width in the plane of the screen, adjusted appropriately for subject’s inter-ocular distance. The star density was 0.01 per cubic cm and the depth of the field was 130 cm. Stars further away from the subject were set to appear smaller as previously described [32]. All conditions were performed in total darkness. Blinders on the sides and top of the screen masked the surrounding walls to avoid platform motion cues. The visual stimulus was presented at a constant velocity.

**Fig 2:**
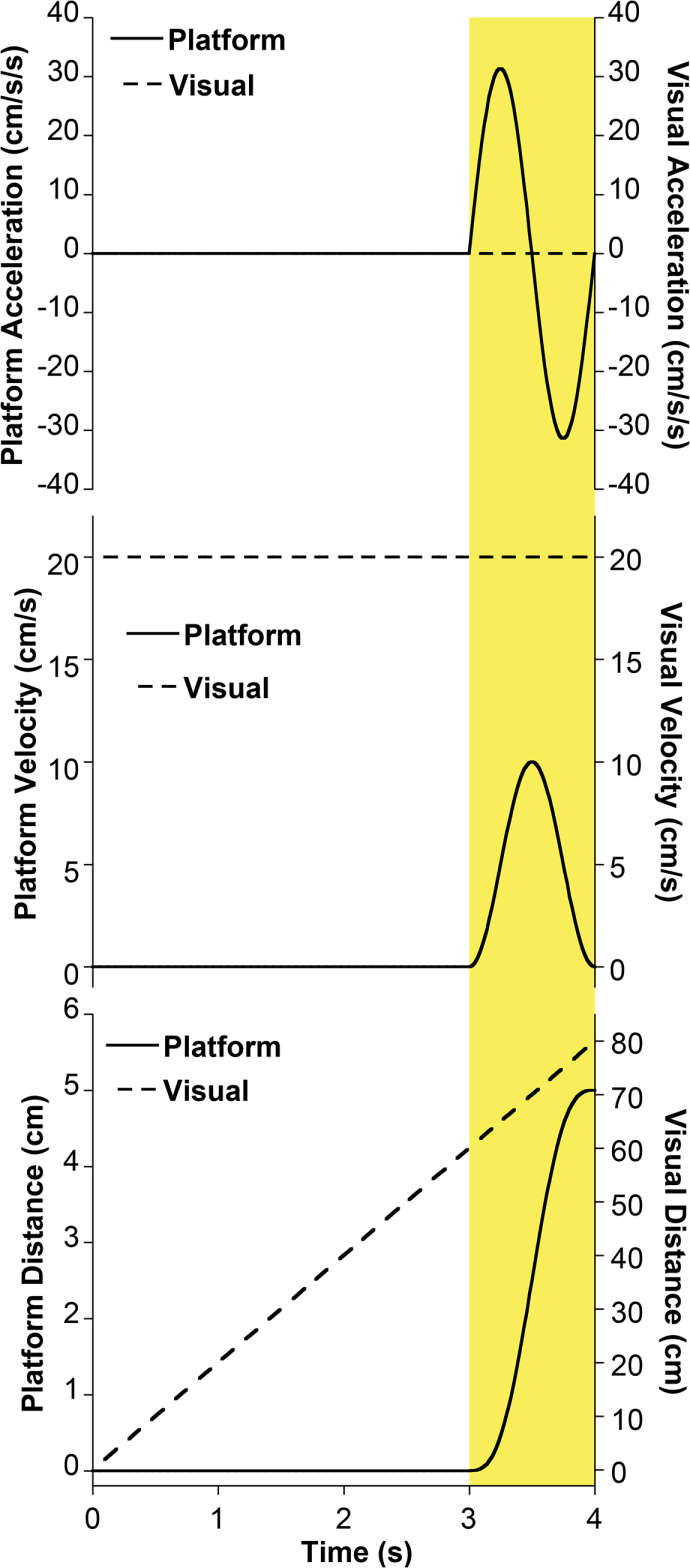
Motion profile corresponding to a trial in which there was 4s of visual motion. Platform motion (solid line) occurred during the final second of the stimulus (shaded yellow). Visual motion (dashed line) occurred at a constant velocity during the stimulus presentation. A relatively large platform motion stimulus (5 cm) as would typically be delivered at the beginning of a trial block is shown in this example.

Platform motion was delivered during the final 1s of PSE trials, and consisted of a sine wave in acceleration with a maximum displacement of 10cm (20cm/s), delivered in the fore-aft direction. The motion was free of discontinuities in acceleration, velocity, or position. A complete motion profile of visual motion and platform motion stimuli is shown (Fig 2).

### General Procedures

Following platform and visual motion, subjects were instructed to indicate the perceived direction of motion (either forward or backward) by pressing the appropriate button with a hand-held button box, making this a single interval, forced choice task. Trial blocks were broken into two to three sessions, each lasting between one and three hours. Trial blocks were randomly chosen, and no set order was created. When participating in the experiment, all subjects were offered breaks between trial blocks to prevent fatigue.

An adaptive staircase was used to determine the point of subjective equality (PSE). The staircase used a one-up, one-down variable step size. Each block was designed to start with a large platform motion stimulus that was likely to be unambiguously perceived, with the stimuli becoming incrementally smaller as the experiment progressed. Two independent staircases were used for each direction of visual motion such that one staircase started with the maximum inertial motion in one direction and the other started with the maximum inertial motion in the opposite direction. Thus, because each trial block included both looming and receding visual motion, 4 randomly interleaved staircases were included with 25 stimulus presentations each (100 total). Staircases were randomly interleaved to decrease the ability of subjects to predict stimuli presentation based on prior experience.

Certainty Estimation (CE) trials were conducted in a similar fashion as the PSE trials, but without platform motion. The same fore-aft visual stimuli were presented. Subjects were instructed to verbally report perceived direction and certainty of self-motion at the conclusion of the stimulus based on a scale of 0 to 100), a scale that has been used successfully in previous work aiming to quantify vection [11,33,34]. Zero was defined as no feeling of motion, and 100 defined as “extremely compelling”, and no additional reference points were suggested as subjects have been found to replace these with their own internal reference values [13].

### Data Analysis

**Fig 3:**
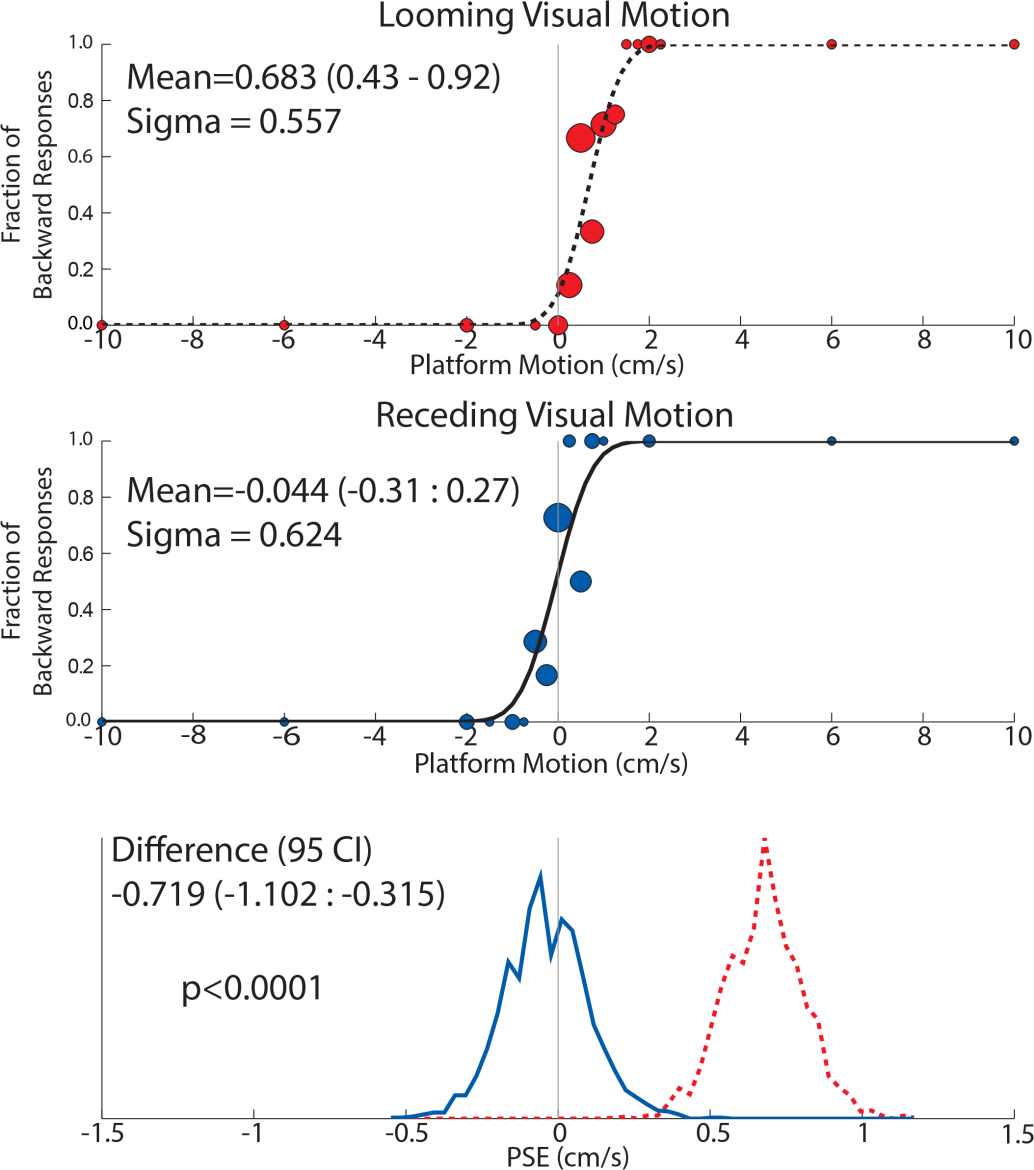
Data from a block of trials in an individual subject (subject 12, from Exp 2). In this block there was a 8s visual stimulus. Circles in the two upper panels are sized proportionally to the number of responses represented. A cumulative distribution function (CDF) was calculated from each data set as a method for determining the mean (bias) and sigma (threshold) of inertial motion detection for each test condition. In this subject, the CDF had a significant shift towards the right when looming VFM was presented (top panel, dashed curve) when compared with receding VFM (middle panel, solid curve). Thus for when there was no inertial motion, looming VFM was reported as forward self-motion. Each CDF was fit to the data 2,000x after being randomly resampled prior to each fit. The histograms of these fits are shown in the bottom panel which demonstrates a significant difference between the two conditions based on no overlap between the curves.

For the PSE trials, the proportion of forward and backward responses was modeled by a cumulative Gaussian function using a Monte Carlo maximum-likelihood criteria as previously described and used in this current laboratory. Data were resampled randomly with replacement to generate multiples estimates of the mean and 95% confidence intervals [31,35,36]. Sample psychometric fitting for a subject is shown(Fig 3). The point of subject equality was defined as the mean of the Gaussian distribution, and represents the motion that elicits responses divided equally between the two possible responses. Deviations from a mean of zero represent an inertial nulling velocity that is equal and opposite to the perceived direction of motion (vection). Threshold was defined as the sigma or width of the cumulative Gaussian distribution. The level of significance in the difference of the means of forward vs backward PSE defined as p < 0.01 [32].

## Experiment 1: Point of Subject Equality vs Certainty Estimates of Vection Perception

### Subjects

We recruited ten adult human subjects (6M, 4F) with no known history of visual or vestibular symptoms. Mean age was 34 ± 15 (mean ± SD; range, 19–60). All subjects underwent general screening for history of dizziness, vertigo, hearing and vision problems, as well as videonystagmography with caloric testing. History of neurologic problems, and rheumatic disease was also explored. Additional demographic information is available in Table 1. Two subjects (Subject 1 and Subject 8) were familiar with the design of the study.

**Table 1:** Demographic data for experiments 1–3. Values in parenthesis represent standard deviation.

**Experiment 1**			
Demographic		Control	Migraine
Age		33.7 (14.7)	37.4 (16.1)
Gender			
	Male	5	4
	Female	0	1
Race			
	White	5	4
	Asian		
	Black		1
Handedness			
	Right	5	5
	Left		
	Ambidextrous		
**Experiment 2**			
Age		25 (6.2)	31 (9.8)
Gender			
	Male	6	2
	Female	2	9
Race			
	White	5	10
	Asian	3	1
	Black		
Handedness			
	Right	7	11
	Left		
	Ambidextrous	1	
**Experiment 3**			
Age		24 (2.5)	24 (4.9)
Gender			
	Male	4	1
	Female	2	5
Race			
	White	4	6
	Asian	2	
	Black		
Handedness			
	Right	6	6
	Left		
	Ambidextrous		
Migraine Type			
	None	6	
	Typical Migraine		6
	Vestibular Migraine		
	Migraine with Aura		2
	Migraine w/o Aura		4

Following the study, additional history was obtained post-hoc regarding migraine. Five subjects met International Headache Society criteria for migraine [37]. None of these subjects met criteria for vestibular migraine [38].

### Procedures

Three sets of control trials were performed to determine baseline bias in visual and motion perception. Baseline inertial bias was measured in the *platform motion control* trial, with platform motion in darkness (no visual motion counterpart). Baseline visual bias was measured in two separate trials: platform motion with a *static visual stimulus* and platform motion with a *zero coherence visual stimulus*. For each trial, a 1s stimulus was presented and the subjected was instructed to indicate the direction of perceived motion. The stimulus was presented to the subject 50 separate times in each control trial. Three separate trials were conducted with stimulus durations of 1, 4, 8, or 16s. Blocks of motion perception trials were presented in random order, but with the 16s trial generally presented last because this condition was added after initial data had already been collected. Platform motion occurred during the final 1 s of the visual stimulus in the forward or backward direction except for CE trials when there was no platform motion.

### Data Analysis

Two-way ANOVA with repeated measures was used for within-subject factors of visual motion direction (two levels: looming and receding) and duration (three levels: 1, 4, and 8s). Pearson’s correlation coefficient was used to test correlation between the PSE and CE trials. Statistical significance was defined as *p* < 0.05. Post-hoc analysis by migraine diagnosis was performed using two-way ANOVA.

### Results

#### Point of Subjective Equality Trials

The experiment was well tolerated and all subjects completed all test conditions. All subjects were able to correctly identify the direction of the inertial stimulus at the start of the staircase (the platform motion stimulus of largest magnitude).

**Fig 4:**
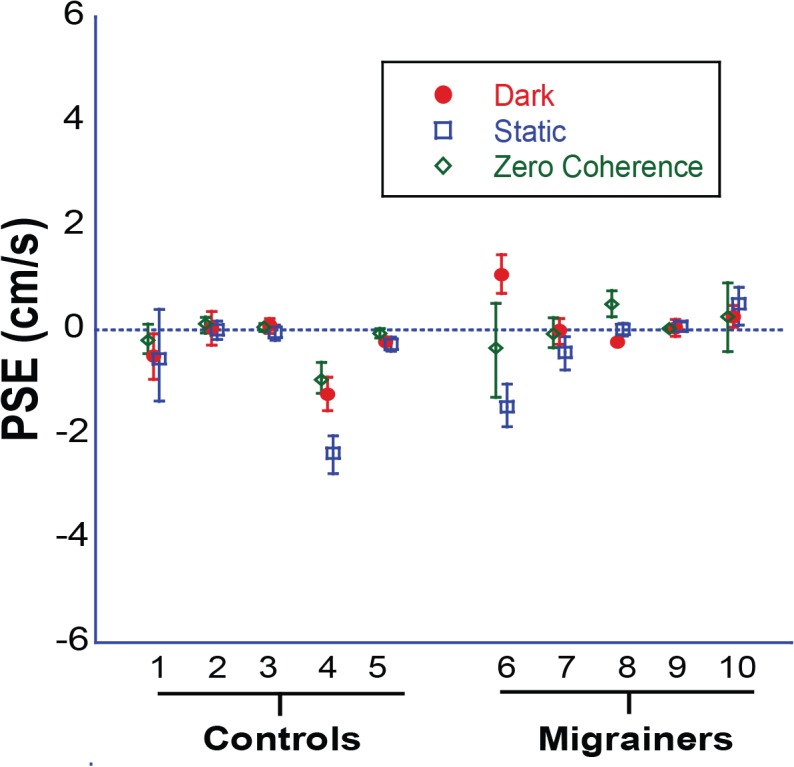
Individual Data for Experiment 1. *Control Trials*. Platform motion in darkness (red circles). Viewing a static visual stimulus (blue squares). Viewing a 0% coherence visual stimulus (green diamonds). Error bars represent 95% CI.

Baseline inertial bias (measured with the *platform motion control* trial), as well as baseline visual bias (measured with *static visual stimulus* and *0% coherence trials)*, are reported (Fig 4). The majority of subjects showed negligible bias in all three control conditions. Two individuals (subjects 4 & 5) had small but consistent backward motion biases, and subject 6 showed varying biases across control conditions. None of the subjects were excluded based on these results.

**Fig 5:**
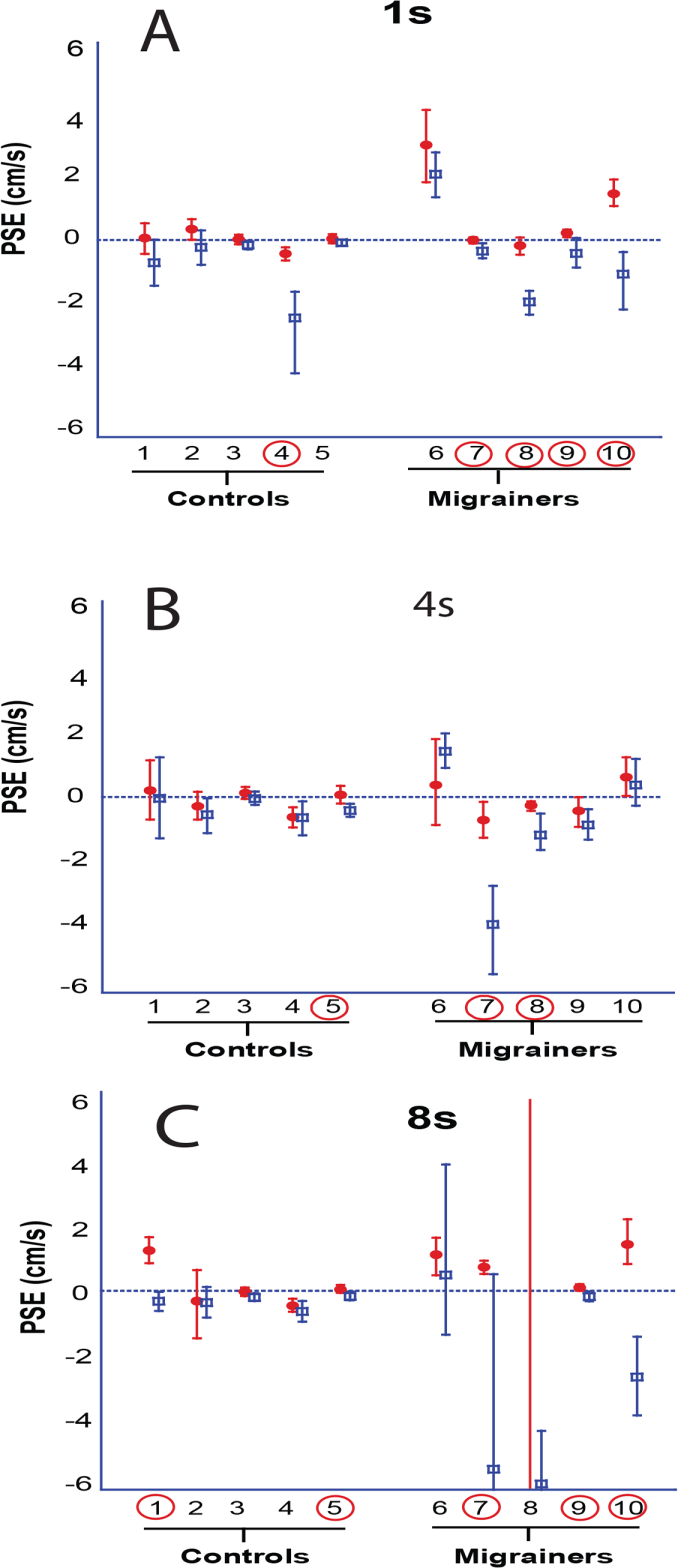
A-C. 1s, 4s, and 8s trials. Looming VFM represented with red circles, receding VFM with blue squares. A positive PSE indicates that a neutral motion would be more likely to be perceived as forward self-motion, likewise a negative PSE indicates a neutral motion would be perceived as backwards. Error bars represent 95% CI.

**Fig 6:**
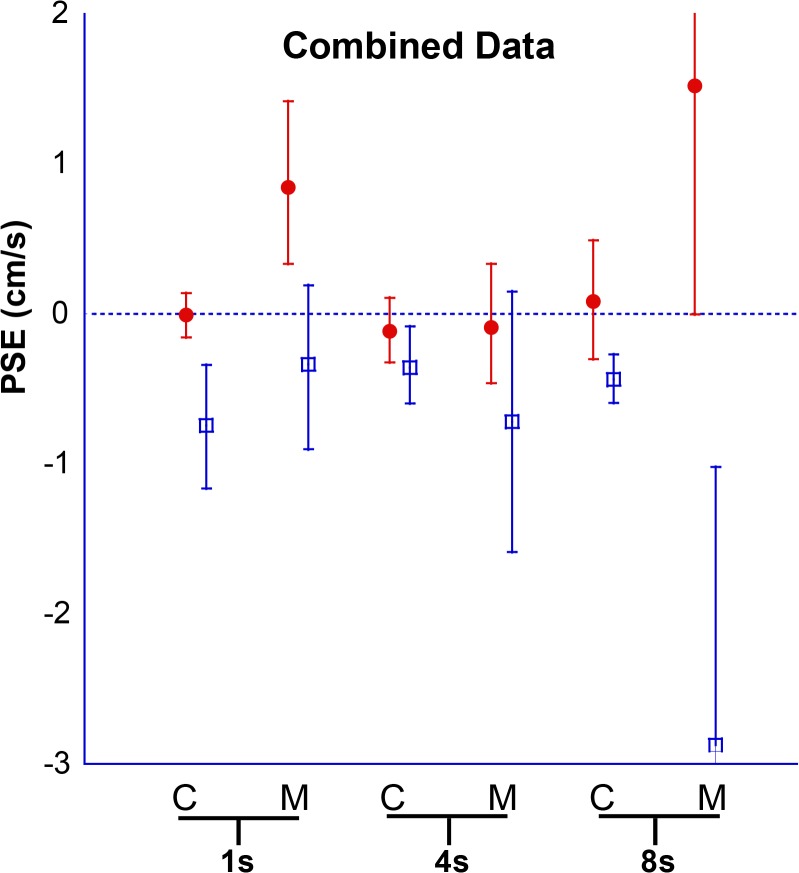
Combined data for PSE at 1s, 4s, and 8s. Looming VFM represented with red circles, receding VFM with blue squares. A positive PSE indicates that a neutral motion would be more likely to be perceived as forward self-motion, likewise a negative PSE indicates a neutral motion would be perceived as backwards. Error bars represent 95% CI.

**Fig 7:**
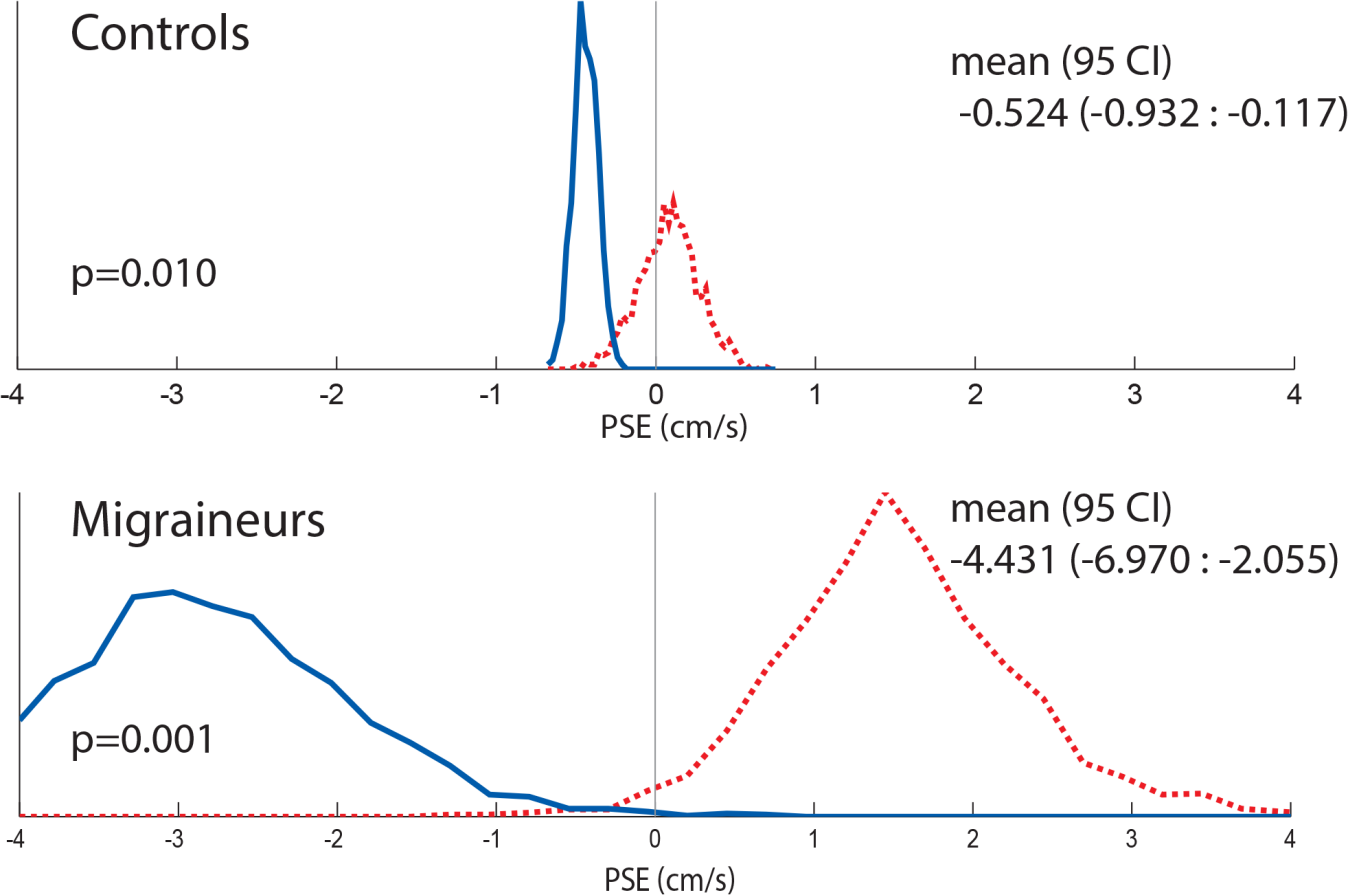
Histogram comparing combined controls and migrainers response using random resampling of responses for 8s visual stimuli. Responses from all subjects were included. Responses collected with looming VFM are represented with a dashed red line, receding VFM responses are represented with a solid blue line.

Results for individual PSE trials based on duration are shown (Fig 5), and combined data are shown (Figs 6 and 7). The interaction of direction and duration on vection was significant (F(2, 36) = 3.97, p = 0.03). The effect of direction of motion on vection was also significant (F(1, 18) = 7.28, p = 0.01). Duration alone did not significantly affect vection experience (F(2,36) = 0.188, p = 0.83).

#### Certainty Estimation (CE) Trials

**Fig 8:**
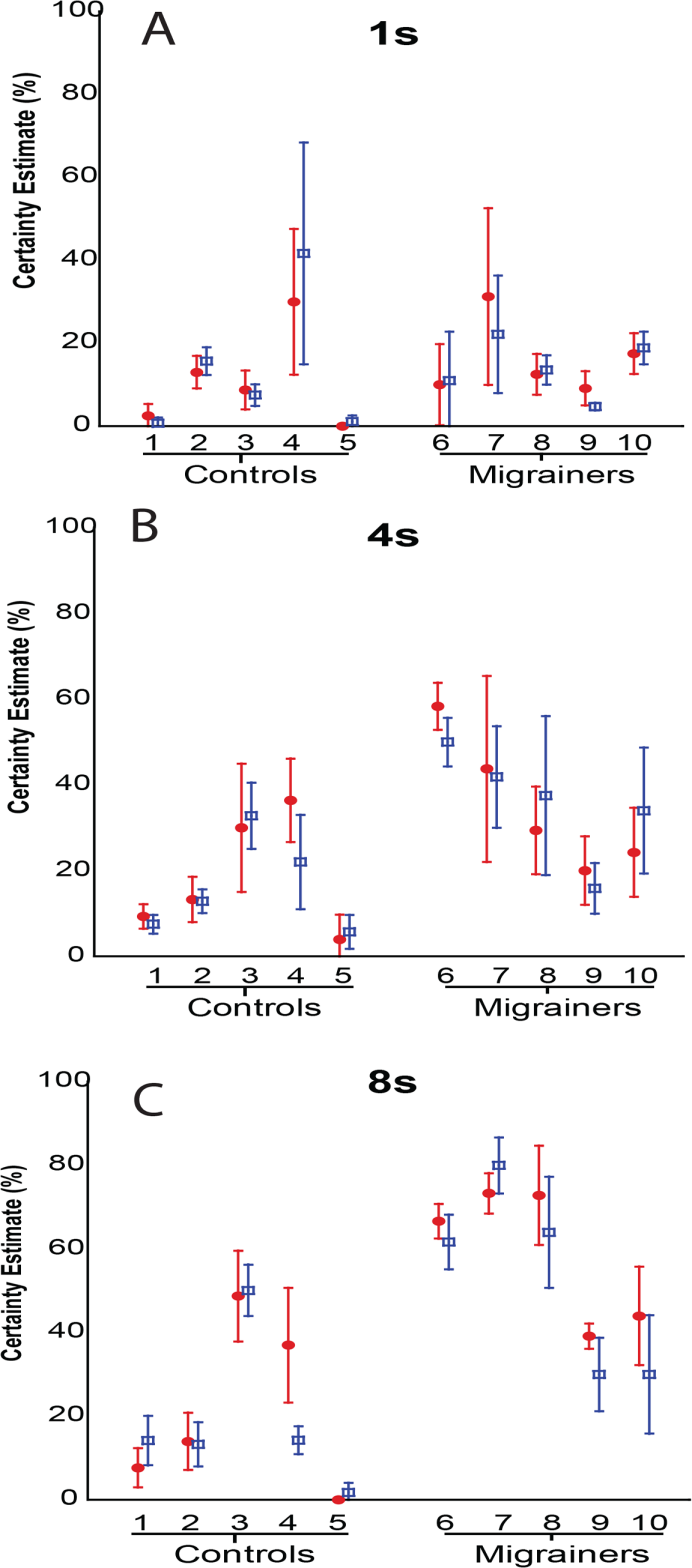
Certainty Estimate–Individual Data for Experiment 1. A-C represent individual data means for 1s, 4s, and 8s trials, respectively. Error bars represent 95% CI.

Subject-reported certainty estimates of self-motion for visual fore-motion trials are shown (Fig 8), Overall, longer durations of visual motion significantly increased the certainty estimate, with significant differences noted between VFM durations of 1s, 4s, and 8s stimuli (One-way ANOVA, F = 5.88, p = 0.004). When comparing CE from looming vs receding visual motion, no significant difference in CE with visual motion direction was found.

#### Point of Subject Equality vs Certainty Estimate Trials

Pearson's correlation coefficient was obtained for each trial and for all trials collectively. The 8s PSE and CE looming and receding VFM studies had a strong correlation of r = 0.48 and r = -0.58, respectively. Correlation between the other studies were not significant, with the exception of the 1s backward stimulus (r = -0.51).

#### Post-Hoc Association of Vection Certainty Estimates with Migraine Diagnosis

Following data collection, each subject completed a questionnaire about personal migraine history, and 5 patients were found to have migraine. Diagnosis of migraine was associated with significantly increased perception of self-motion by CE (Two-way ANOVA repeated measure, *F*(1,18)) = 7.29, *p* = .01) but not IN (ANOVA, *F*(2/32), *N*.*S*.).

## Experiment 2: Effect of Migraine on Vection Perception

The results from Experiment 1 suggest that migraine may sensitize subjects to vection. In Experiment 2, we prospectively enrolled subjects with and without a history of migraine to determine the impact of migraine diagnosis on vection.

### Design and Methods

#### Subjects

We recruited 18 adult human subjects (8F, 10M) with no known history of vestibular or vision disorders besides migraine or vestibular migraine. Mean age was 28 ± 8.9 (mean ± SD; range, 18–49). A detailed history of migraine and vestibular migraine was obtained and diagnosis of migraine verified using the International Headache Society criteria. All subjects underwent general screening for history of dizziness, vertigo, hearing and vision problems, and a history of neurologic problems and rheumatic disease was also explored. Additional demographic information is available in Table 1. Two subjects (subjects 8 and 16) had also performed Experiment 1.

#### Equipment and Stimuli

The equipment and stimuli were as for Experiment 1, with three minor changes. The velocity of the visual fore-motion stimulus was increased to 50cm/s from 20cm/s. The star density was decreased to 0.0035 per cubic cm in order to optimize the visual stimulus and the certainty estimate trials consisted of two trials of each stimulus instead of three.

### Results

The experiment was well tolerated and all subjects completed all test conditions. During PSE trials, all subjects were able to correctly identify the direction of the inertial stimulus at the start of the staircase (the platform motion stimulus of largest magnitude).

#### Point of Subjective Equality Trials

**Fig 9:**
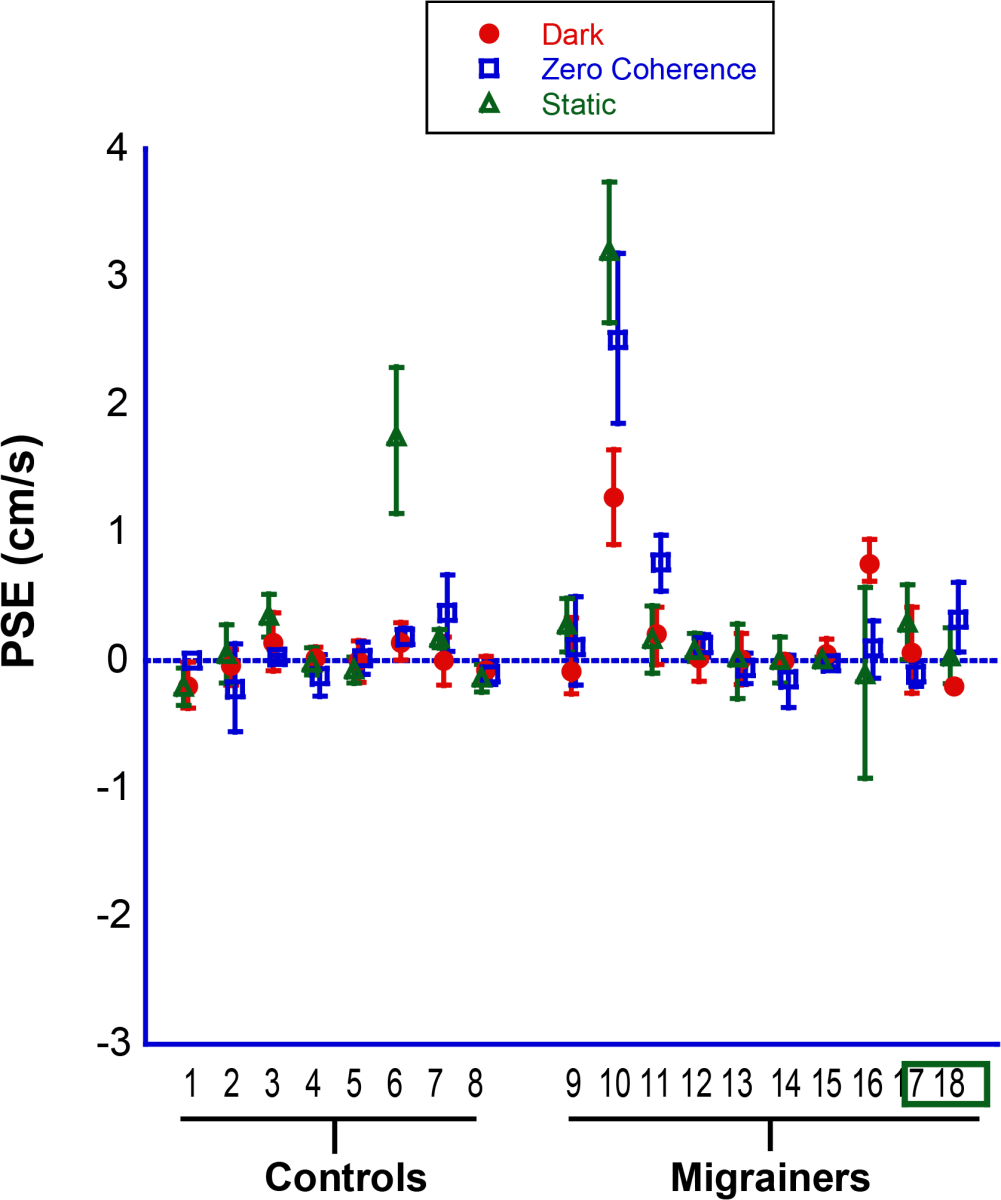
- Individual Data for Experiment 2. *Control Trials*. Platform motion in darkness (red circles). Viewing a static visual stimulus (green triangles). Viewing a 0% coherence visual stimulus (blue squares). Error bars represent 95% CI.

Baseline inertial and visual bias was tested with three controls (as above), and are reported (Fig 9). Subject 8 and 10 had consistently positive PSE during control tests.

**Fig 10:**
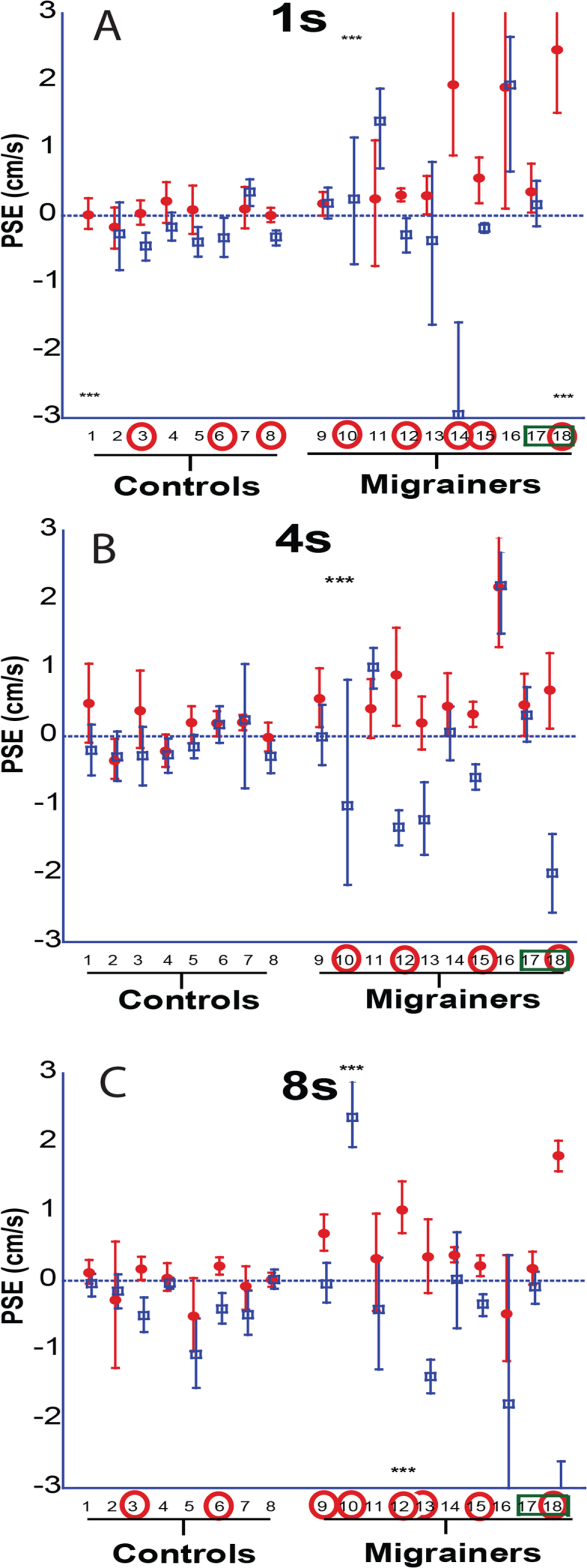
A-C. 1s, 4s, and 8s trials. Looming VFM represented with red circles, receding VFM with blue squares. Subjects marked with a ‘***’ indicate that one of the data points exceeds the limits of the plot. The mark is shown at the top if the plot of the data point exceeds the positive limits and at the bottom if it exceeds the negative limits. Error bars represent 95% CI.

**Fig 11:**
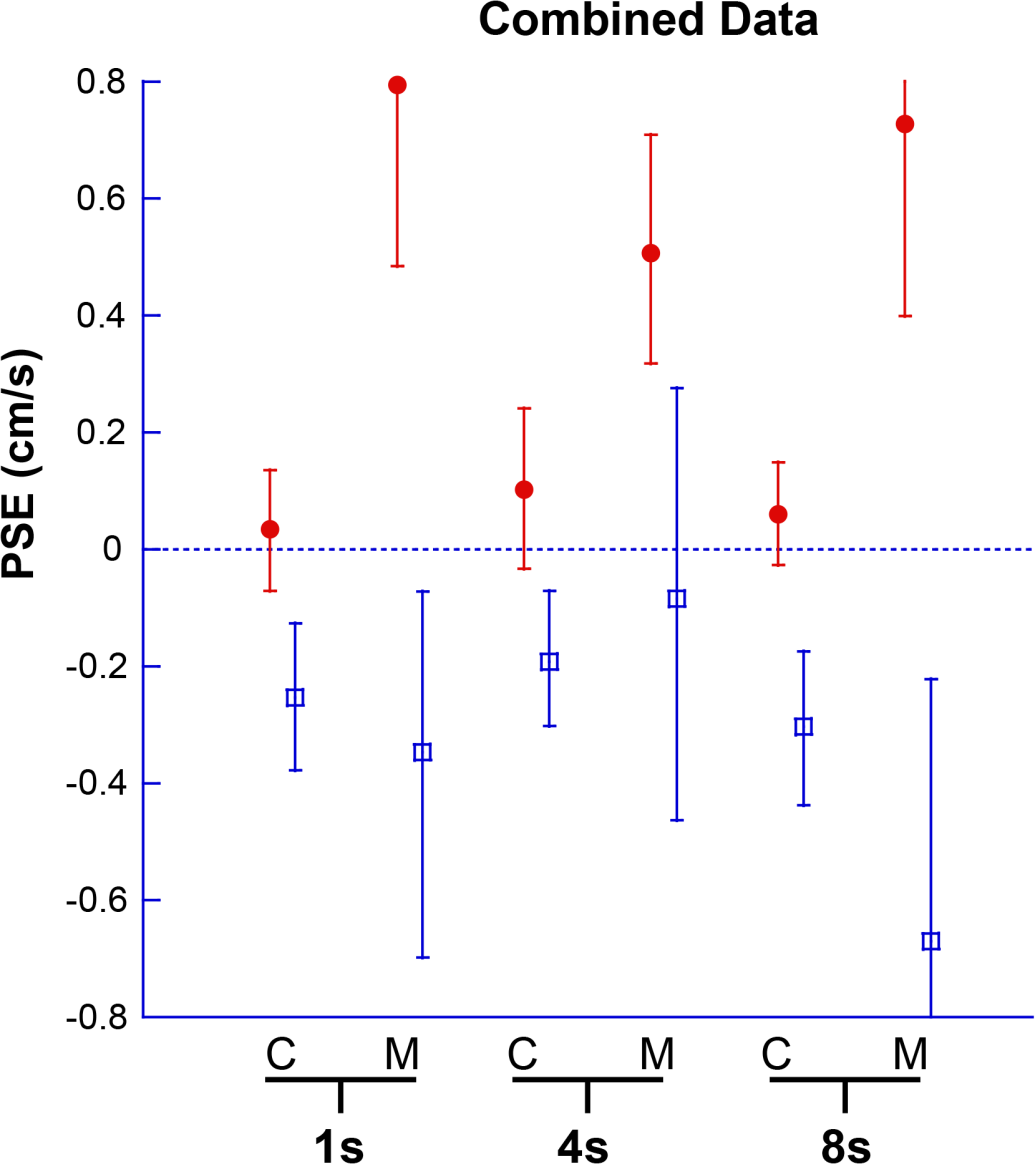
Combined Data for Experiment 2. Looming VFM are represented with filled red circles, receding VFM are represented by blue open squares. A positive PSE indicates that neutral motion would be perceived as forward self-motion. Error bars represent 95% CI.

**Fig 12:**
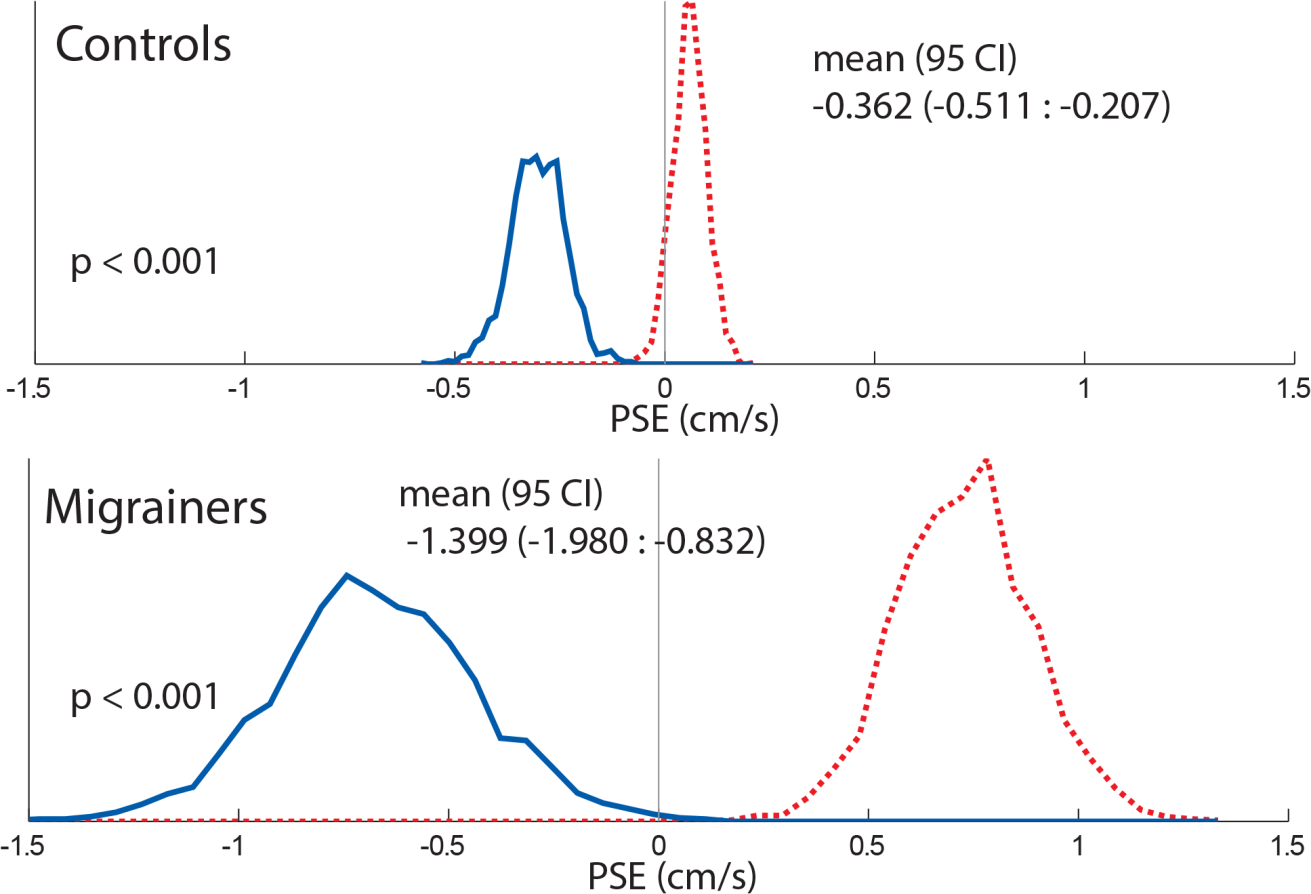
8s Histogram comparing combined controls and migrainers response using a random resampling of responses. Responses from all subjects were included. Responses collected with looming VFM are represented with a dashed red line, receding VFM responses are represented with a solid blue line.

The PSE was determined for 1s, 4s, and 8s stimuli (Fig 10), and combined data are also shown (Figs 11 and 12). Migraine subjects required a greater velocity to null their perception of motion in the 8s stimulus (mean difference in PSE = 1.48 cm/s, student t-test, p = 0.04). At 1s and 4s, there was no significant difference between migraine and control.

#### Certainty Estimate Trials

**Fig 13:**
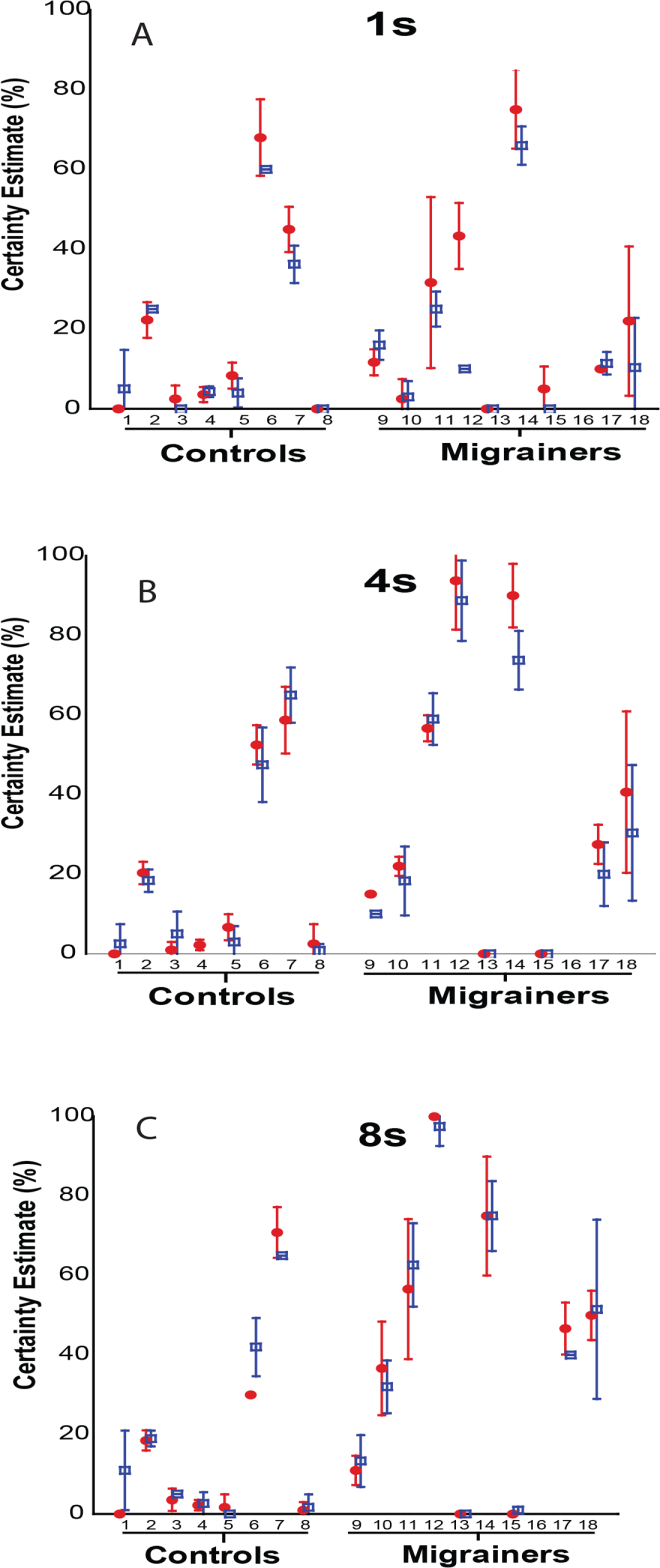
Certainty Estimate—Individual Data for Experiment 2. A-C represent individual data means for 1s, 4s, and 8s trials, respectively. 8 stimuli were delivered in each trial as either the looming or receding VFM. Mean certainty estimates for each subject were collected with looming VFM represented with a red solid circle, receding VFM responses are represented with a blue square. Error bars represent 95% CI.

Subject-reported certainty estimates of self-motion for visual fore-motion trials are shown (Fig 13). Diagnosis of migraine was associated with significantly increased perception of self-motion by CE across the three VFM studies (Two-way ANOVA, *F*(1,101)) = 4.251, *p* = .04).

## Experiment 3: Effect of Migraine and Long-Duration Visual Motion Stimuli on Vection Perception

Experiment 2 demonstrated significant differences between subjects with migraine and control subjects in the 8s duration trials. We hypothesized that longer duration visual stimuli might be more likely to produce a perception of vection, especially in subjects with Migraine. Some past studies have shown a mean onset of vection at greater than 10s [1,11]. Experiment 3 explores vection perception for subjects with and without migraine for 16 s long visual stimuli.

### Design and Methods

#### Subjects

We recruited 12 subjects (7F, 5M), 8 of whom had participated in Experiment 2 of the study. All subjects were screened similar to Experiment 2. Mean age was 24 ± 3.7. Six subjects had migraine and six did not. One subject with migraine was unable to complete the task and was excluded from the study. Demographic information is available in Table 1.

Equipment and stimulus, as well as PSE and CE trial designs, were identical to Experiment 2 of this study.

### Results

The experiment was again well tolerated and all but one subject completed all trials. All subjects were able to correctly identify the largest (initial) stimulus.

#### Point of Subjective Equality Trials

**Fig 14:**
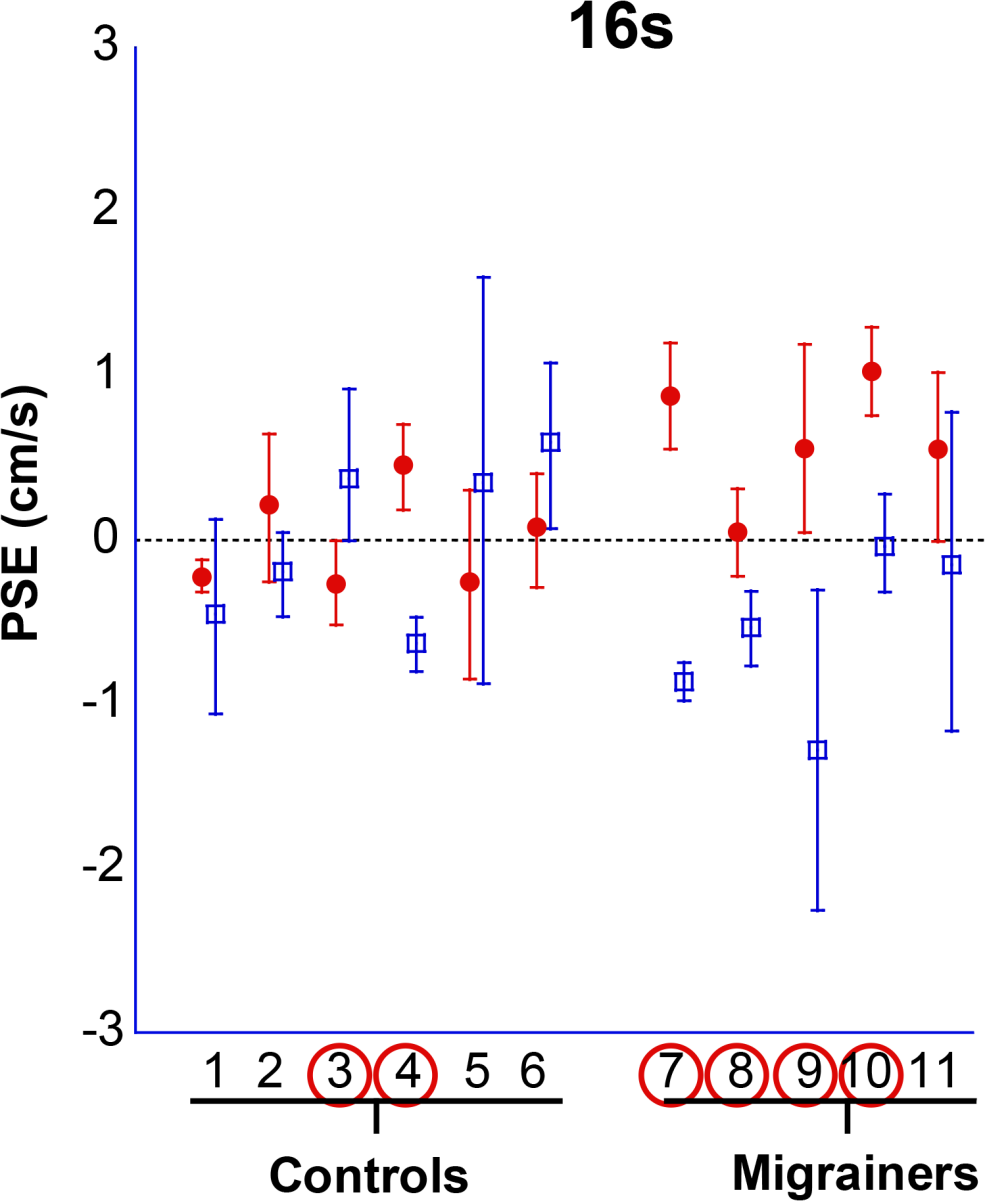
16s duration VFM stimulus. Individual subject PSE for inertial nulling of visual field motion. Subjects are represented along the x-axis (subjects 1–11). Looming VFM trials are represented by solid red symbols; receding VFM trials by open blue symbols. A positive PSE indicates a stationary platform would be likely to be perceived as forwards self-motion. Circled subject numbers indicate a significant difference between these (p < 0.05). Error bars represent 95% CI.

**Fig 15:**
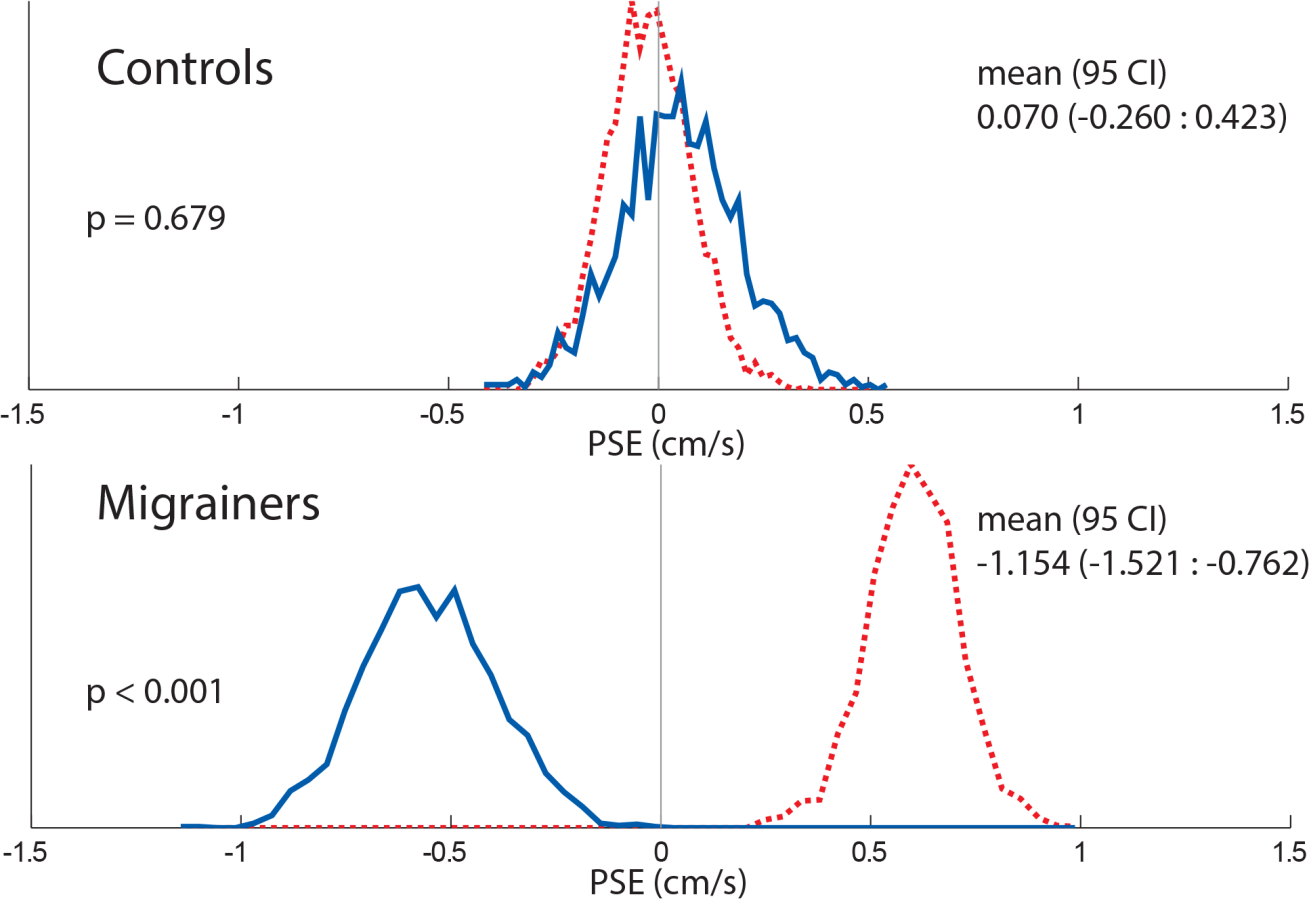
16s Histogram comparing combined controls and migrainers response using a random resampling of responses. Responses from all subjects were included. Responses collected with looming VFM are represented with a dashed red line, receding VFM responses are represented with a solid blue line.

The PSE was determined for each subject along with 95% confidence intervals (Fig 14), and combined data (Fig 15). Migraine subjects required a nulling velocity of 1.18 ± 0.51 cm/s to null their perception of motion, which was statistically greater than controls (PSE = -0.008 ± 0.56 cm/s, p = 0.01).

#### Certainty Estimate Trials

**Fig 16:**
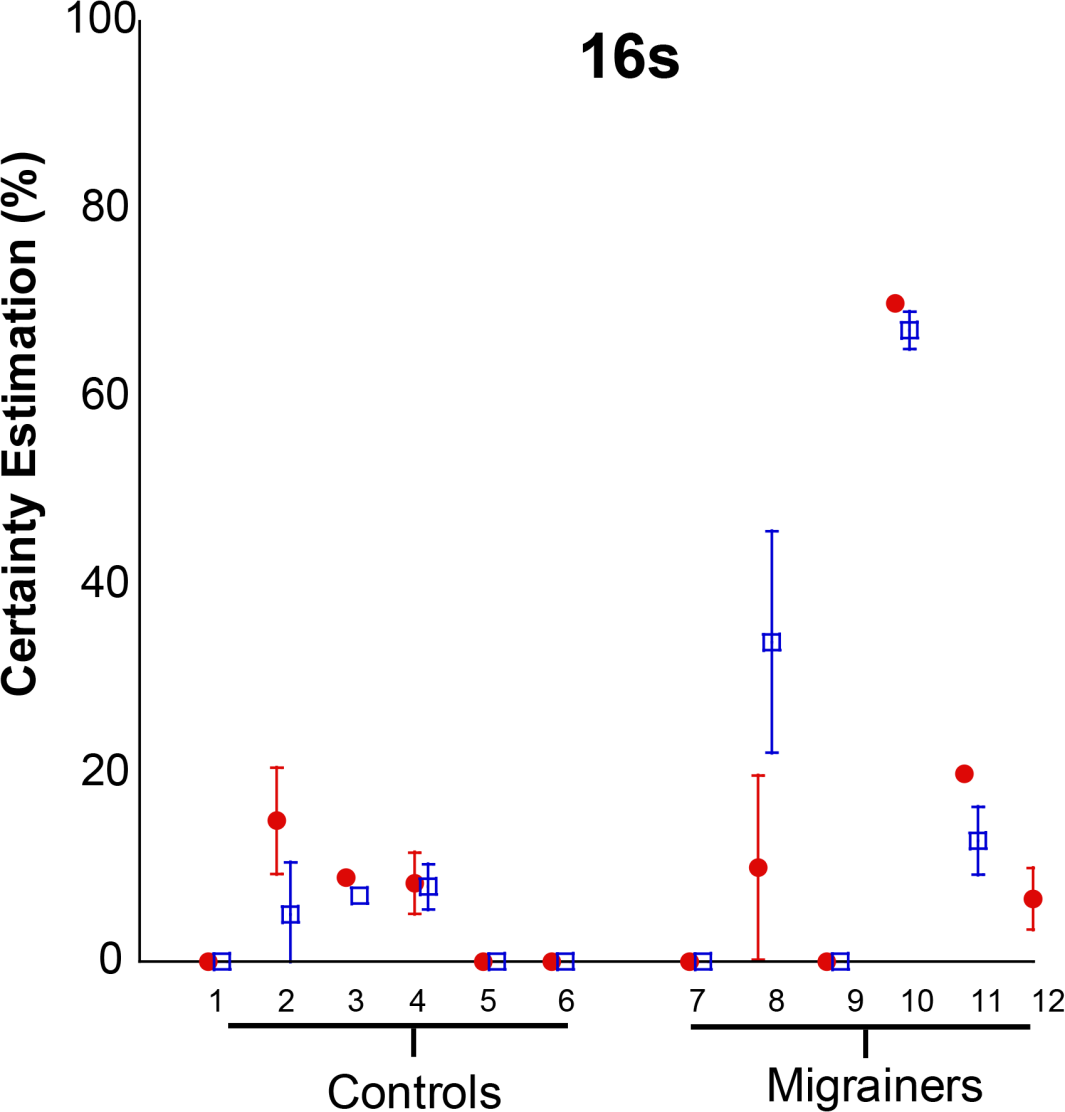
16s Certainty Estimate—Individual Data for Experiment 3. 8 stimuli were delivered in each trial with either the looming (red circles) or receding (blue squares) VFM. Mean certainty estimates are shown with error bars representing 95% CI.

Subject-reported certainty estimates for the 16s VFM are shown (Fig 16). Migraine subjects reported greater subjective feeling of motion (CE = 24.6 ± 6.96) than controls (CE = 5.4 ± 1.53, p = 0.0002).

## Discussion

The current study examined vection induced by the same VFM stimulus using two different measures. Experiment 1 directly compared vection measurements made using magnitude estimation and inertial nulling techniques. Although measurement using an inertial stimulus has been previously attempted, these studies demonstrated substantial individual variation and did not directly compare IN methods and subjective methods [12,18,19]. The current study demonstrates a strong correlation between vection measured using the CE and IN at 8s duration.

Our study still demonstrated interesting differences between CE and IN. For example, subjects 13 and 15 in Experiment 2 had significant vection in the IN techniques, but both subjects reported zero perception during their CE trials at 8s. Given the same instructions, subjects can vary in their internal interpretation of the scale, and subject 13 and 15 may have chosen zero as a response because they knew that the platform was not going to move and chose to focus on that during their response, or indeed to have no perception of self motion. With the inertial nulling technique, subjective reporting is minimized by using a forced-choice task. And since each subject undergoes the same forced-choice task, the ability to accurately interpret the subjective experience of vection is enhanced.

Nearly half of all subjects did not have measurable vection by inertial nulling at 8s and 16s in our study [39]. Defining a cut-off for determining what is truly vection using certainty estimate is ambiguous and difficult, but the data show similar numbers for certainty estimate. Other studies using IN methods also reported a subset of subjects who experienced little or no vection or subjects who were not able to do the task which might have been due to no vection being perceived [12,18]. Still other studies have avoided this problem altogether by reporting aggregate data. Unfortunately, this makes it impossible to determine the degree of within- and between-subject variation in perception [9–11,16,40,41]. Although maximizing vection was not the goal of this study, modifications of the stimulus parameters could be made to create a more compelling stimulus. Notwithstanding, the current stimulus still created a motion experience that allowed for correlations to be made between the measurement devices, and for analysis of two separate populations to be made.

Throughout this study we have compared CE and IN techniques for measuring vection. Since IN involves a physical motion and CE does not, one could raise the point that PSE measurements are actually measuring visual-vestibular integration, and not vection which is purely visual [42,43]. Our 1s IN trials consist solely of an overlapping visual and platform motion. While initially designed to be a control (wherein no vection is experienced), we did indeed find significant variation in some subjects, particularly in migraine subjects (Fig 10), and this finding does suggest difficulty with visual-vestibular integration. However, the effects of the 1s overlapping stimulus does not mirror that of the 4s and 8s stimuli in controls and migraine subjects. Furthermore, CE trials seem to correlate with the IN measurements at durations that produce vection (>6–8s). We believe that during the longer stimuli, vection is being produced, and that vection is being measured by a nulling velocity that counteracts the already present illusion of self-motion.

It should be noted that the masking sound creates a looming sound suggesting that one approaches and then retreats from the source of the noise. This might create an auditory illusion of self-motion and could have affected our results [44]. That said, the masking sound was uniform in all trials, and could not explain the difference in populations. Furthermore, the sound was present in the dark trials of certainty estimate, where there was no motion or visual stimuli. The average CE response in Experiment 2 when the only stimulus was the masking sound was 0.61% out of 100, with no subjects reporting a CE greater than 5% at any point, highlighting the insignificance of this masking sound.

One important consideration for the observed variation in vection among studies may be the lack of screening for migraine diagnosis. A major finding of this study is that migraine subjects demonstrated larger vection measurements in both the CE and IN trials. Given that the prevalence of migraine in the population is almost 18% in females, it is likely that other studies of vection perception included some individuals with migraine [45].

Migraine is a broad diagnosis that applies to several different presentations. Migraine patients often experience vestibular symptoms, such as vertigo and the feeling of tilting during migraine episodes [20,23]. It has frequently been observed that motion sickness is a common feature in migraine and specifically visually induced motion sickness (VIMS) [21,22,25–29]. VIMS has been described in a context of visual motion [46–48]. It has also been hypothesized that vection is required for a VIMS to be induced [49–51], Since the link between VIMS and vection has already been established, this provides a plausible mechanism by which VIMS may occur in migraine. To our knowledge this is the first study that suggests a link between vection perception and migraine.

The diagnosis of migraine did not uniformly affect results, as some migraine subjects had minimal vection using one or both measurement techniques. It is difficult to determine the cause of this variation. Variations in migraine presentation (aura vs no aura), severity or frequency, and location make finding a homogenous migraine population difficult. Much larger studies would be needed to compare migraine by these subcategories.

Our data demonstrate a strong correlation between vection measured by CE and IN methods. Point of subjective equality through inertial nulling is a novel technique that may enhance the study of vection by minimizing the subjective nature of reporting, and allowing for intra- and inter-subject analysis. Using both tools, we demonstrated that migraine subjects tended to have an enhanced vection that may account for some of the variability in vection perception. Migraine should be appropriately screened for and noted in future studies of vection.
